# A snapshot study of the microbial community dynamics in naturally fermented cow’s milk

**DOI:** 10.1002/fsn3.2174

**Published:** 2021-02-16

**Authors:** Wei‐Liang Xu, Chun‐Dong Li, Yuan‐Sheng Guo, Yi Zhang, Mei Ya, Liang Guo

**Affiliations:** ^1^ Xilingol Vocational College Xilin Gol Institute of Bioengineering Xilin Gol Food Testing and Risk Assessment Center Xilinhot China

**Keywords:** bacteria, fungi, microbial dynamics, natural fermentation

## Abstract

Natural fermentation of milk is a prerequisite in the production of traditional dairy products and is considered a bioresource of fermentative microorganisms and probiotics. To understand the microbial dynamics during distinct fermentative phases, the roles of different microbes, and the relationship between bacteria and fungi, microbial community dynamics was investigated by culture‐dependent and culture‐independent approaches. Natural, static fermentation of milk induces the formation of the underlying curds and the superficial sour cream (Zuohe in the Mongolian language). From an overall perspective, viable LAB increased remarkably. Yeast showed an initial increase in their abundance (from 0 hr to 24 hr), which was followed by a decrease, and mold was detected at the later stages of fermentation (after 68 hr). The observed trends in microbiota variation suggest an antagonistic interaction between bacteria (LAB) and fungi (yeast and mold). The beneficial bacterial and fungal genus and species (e.g., *Lactococcus*, *Streptococcus*, *Leuconostoc*, *Dipodascus*, *Lactococcus lacti*, *Dipodascus australiensis*) are gradually increased in concentration, and the potentially detrimental microbial genus and species (e.g., *Acinetobacter*, *Pseudomonas*, *Fusarium*, *Aspergillus*, *Mortierella*, *Acinetobacter johnsonii*, *Fusarium solani*) decrease during the decline of bacterial and fungi diversity from natural fermentation. The study of microbial community dynamics could make a great contribution to understand the mechanism of natural fermentation of milk and the formation of curds and Zuohe, and to discover the potentially fermentative microbes for industrial starter cultures.

## Graphical abstract

This study is the first to investigate the microbial community dynamics of natural, static fermentation of milk by culture‐dependent and culture‐independent approaches. Our results demonstrated that LAB, yeast, and mold changed dynamically and exhibited the antagonism between bacteria (LAB) and fungi (yeast and mold). The beneficial bacterial and fungal genus and species are gradually increased in concentration, and the potentially detrimental microbial genus and species decrease during the decline of bacterial and fungi diversity from natural fermentation.

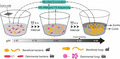

## 1 INTRODUCTION

Milk natural fermentation is an ancient and traditional process that uses raw milk to ferment spontaneously. Traditional dairy products from natural fermentation of milk have always played relevant roles in the nutrition of nomad communities worldwide. Milk natural fermentation was originally developed by nomad Mongolian families, which usually placed the fermentation container into a cold storage room (below 20℃), to stimulate flavor production. Flavor diversity in naturally fermented milk results from increased metabolite production by fermentative bacteria and fungi.

Several naturally fermented milk products derived from livestock of different countries have been investigated in terms of their production process (Guo, Xu, et al., 2019; Yamei et al., 2019), physicochemical composition (Bornaz et al., 2010; Guo, Xu, et al., 2019; Guo, Ya, et al., 2019; Yamei et al., 2019), microbial community (Akabanda et al., 2013; Gao et al., 2017; Gesudu et al., 2016; Guo, Ya, et al., 2019; Liu et al., 2015; Mathara et al., 2004; Nahidul‐Islam et al., 2018; Oki et al., 2014; Shangpliang et al., 2017, 2018; Sun et al., 2014; Takeda et al., 2011; Yamei et al., 2019; Yao et al., 2017; Yu et al., 2011), and health benefits of such microorganisms (Karami et al., 2017; Takeda et al., 2011; Wang et al., 2016, 2018; Yi et al., 2016), such as traditional dairy products in China (Gao et al., 2017; Gesudu et al., 2016; Guo, Ya, et al., 2019; Sun et al., 2014; Wang et al., 2016, 2018; Yamei et al., 2019; Yao et al., 2017; Yi et al., 2016), Mongolia (Oki et al., 2014; Sun et al., 2014; Takeda et al., 2011; Yao et al., 2017; Yu et al., 2011), Russia (Liu et al., 2015), Iran (Karami et al., 2017), India (Shangpliang et al., 2018), Bangladesh (Nahidul‐Islam et al., 2018), Tunisia (Bornaz et al., 2010), Bhutan (Shangpliang et al., 2017), Kenya (Mathara et al., 2004), and Ghana (Akabanda et al., 2013) (Table S1). However, few studies addressed the completely natural static fermentation, the intermediate process for the production of cheese and Zuohe (traditional sour cream). The sour cream moves upwards during natural fermentation of milk to form Zuohe, which is regarded as a Mongolian nutritional dairy product. Furthermore, previous research has been mostly focused on the microbial community of the final products from natural fermentation (Akabanda et al., 2013; Gao et al., 2017; Guo, Ya, et al., 2019; Liu et al., 2015; Mathara et al., 2004; Nahidul‐Islam et al., 2018; Oki et al., 2014; Shangpliang et al., 2017, 2018; Sun et al., 2014; Takeda et al., 2011; Yamei et al., 2019; Yao et al., 2017; Yu et al., 2011), neglecting the dynamics of the microbial community during natural fermentation.

In this study, the microbial community, including LAB, yeast, and mold, during the process of natural fermentation of cow's milk were investigated by using culture‐dependent methods and high‐throughput amplicon sequencing including 16S rRNA and internal transcribed spacer (ITS) to understand the detailed fermentation process, the roles of distinct fermentative microorganisms, and the interplay between bacteria and fungi.

## 2 MATERIALS AND METHODS

### 2.1 Construction of the Natural Fermentation Model and Quantification of LAB, Yeast, and Mold

**FIGURE 1:**
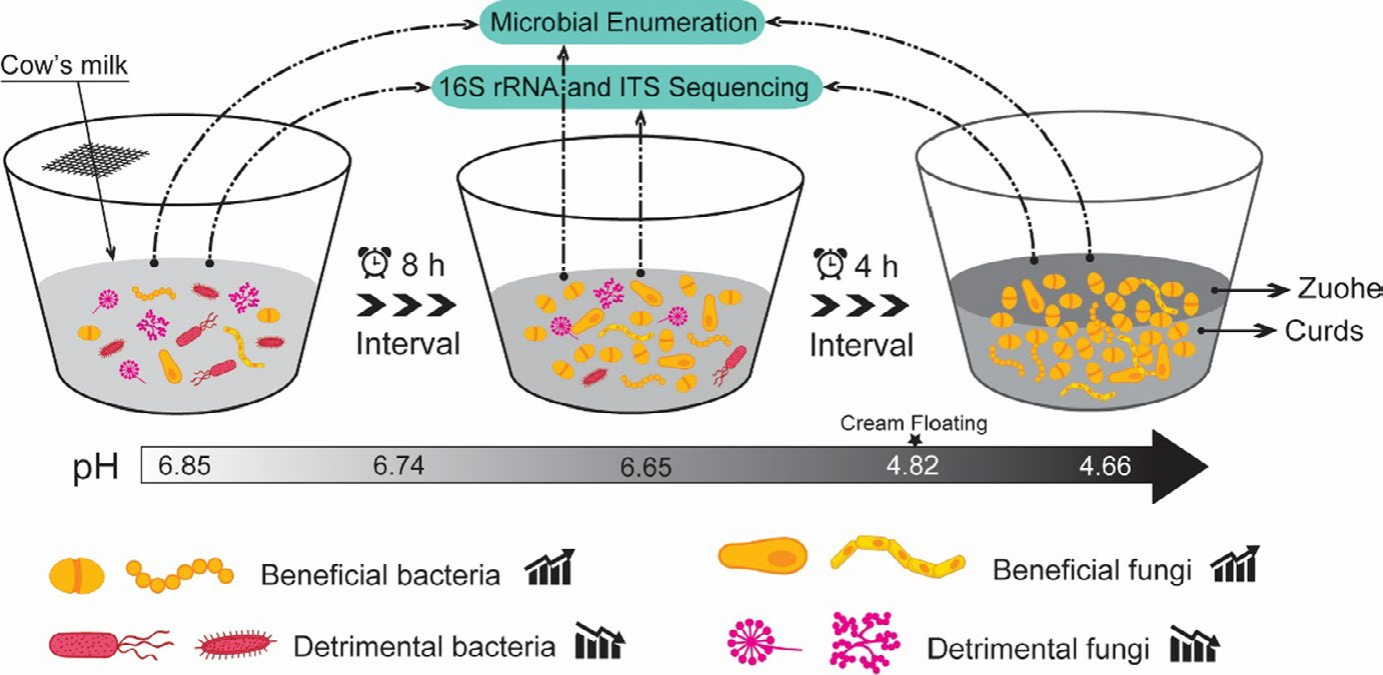
Schematic representation of static, natural fermentation of milk. 100 kg of raw cow's milk was naturally fermented at room temperature (6.3 to 16.7℃), and microbial community dynamics (44 samples) was investigated by using culture‐dependent methods and high‐throughput amplicon sequencing

To simulate natural milk fermentation, 100 kg of raw milk of Holstein from Xilingol prairie was used to ferment at low temperature (6.3 to 16.7℃) (Figure 1). We hypothesized that the fermentation of large amounts of raw milk could stabilize microorganism dynamics, which makes the results more representative. Large‐scale, low temperature natural fermentation was established for investigating bacterial and fungal community dynamics in details, which helps us further understand the changes and relationships of LAB, yeast, and mold in the process of natural fermentation. The protein, fat, and lactose content of raw cow's milk was 3.27%, 3.88%, and 4.92%, and the acidity and pH were 15°T and 6.85. We maintained the temperature of fermentation below 20℃ to extend the period of natural fermentation, allowing a detailed study of microbial dynamics during the process. We clearly found that the viable count of LAB reached its highest value after 92‐hr fermentation, and the pH fell to 4.8 which induced to the curding of milk. Based on these reasons, the 96‐hr fermentation time was determined to be the end of natural fermentation in this snapshot study. The sampling intervals range from 8 hr to 4 hr (0 hr, 8 hr, 16 hr, 24 hr, 32 hr, 36 hr, 40 hr, 44 hr, 48 hr, 52 hr, 56 hr, 60 hr, 64 hr, 68 hr, 72 hr, 76 hr, 80 hr, 84 hr, 88 hr, 92 hr, 96 hr), and the underlying curds and superficial Zuohe (traditional sour cream) were collected to investigate bacterial and fungi community dynamics by using culture‐dependent methods and high‐throughput amplicon sequencing (16S rRNA and ITS). LAB (China National food safety standard, 2016a) were quantified using Man Rogosa Sharp (MRS) for 72 hr at 36°C according to national food safety standards in China. The yeast and mold (China National food safety standard, 2016b) were enumerated using Rose Bengal Agar for 5 d at 28°C.

### 2.2 16S rRNA and ITS sequencing, bioinformatics, and statistical analysis

Microbial DNA was extracted using E.Z.N.A stool DNA kit (Omega Bio‐Tek, Norcross, US). The 16S rRNA V3‐V4 region was amplified with the following primers: 341F 5'‐ CCTACGGGNGGCWGCAG ‐3' and 806R 5'‐ GGACTACHVGGGTATCTAAT ‐3'. The ITS sequence was amplified with the following primers: ITS3‐KYO2F 5'‐ GATGAAGAACGYAGYRAA ‐3' and ITS4R 5'‐ TCCTCCGCTTATTGATATGC ‐3'. The PCR consisted of 5 μl of 10 X KOD buffer, 1 μl of KOD polymerase, 5 μl of 2.5 mM dNTPs, 1.5 μl of each primer (5 μM), and 100 ng of microbial DNA. The thermal program of the reaction was set up as follows: 1 × (95℃ for 2 min), 27 × (98℃ for 10 s, 62℃ for 30 s, 68℃ for 30 s), 1 × (68℃ for 10 min). The amplicon was quantified and subjected to paired‐end sequencing (2 × 250) by the Illumina MiSeq platform (Illumina, San Diego, CA). High‐quality clean reads were obtained by removing reads with more than 10% of unknown nucleotides and less than 80% of bases with quality (Q‐value) > 20. The final effective reads were acquired by removing chimeric tags and were clustered into the operational taxonomic units (OTU) of ≥97% similarity using the UPARSE pipeline (Edgar, 2013). The OTU was classified into organisms by the Naive Bayesian Model using RDP classifier (Wang et al., 2007) based on SILVA database for 16S rRNA sequencing (Pruesse et al., 2007) and UNITE database for ITS sequencing (Koljalg et al., 2005).

## 3 RESULTS AND DISCUSSION

### 3.1 Viability changes in LAB, yeast, and mold during natural fermentation of milk

**FIGURE 2:**
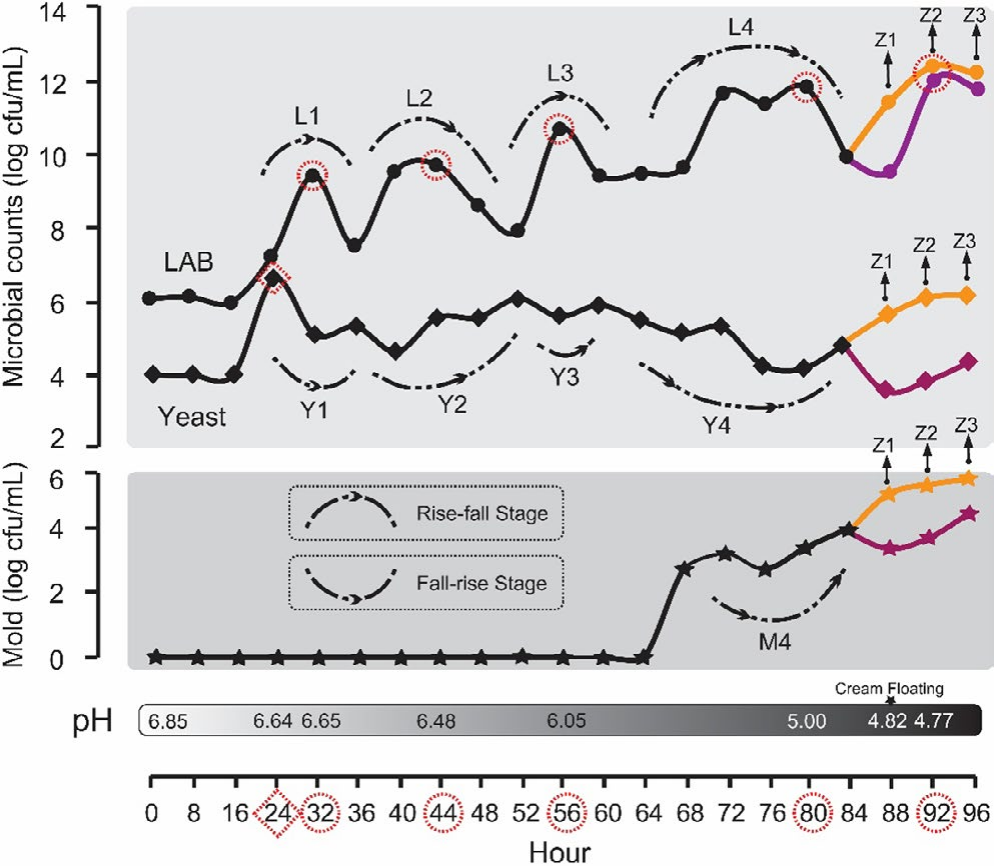
Microbial dynamics during natural fermentation of milk. LAB, yeast, and mold of samples from the continuous fermentation process were quantified. The dynamics of this process included four rise and fall stages of LAB (L1, L2, L3, and L4), four fall and rise stages of yeast (Y1, Y2, Y3, and Y4), and one fall and rise phase of mold (M4)

The total number of LAB and yeast in raw milk at the beginning of the fermentation process were 6.22 log cfu/ml and 4.16 log cfu/ml, respectively. In contrast, we did not detect any mold at this stage of the process. Given the small changes in the microbial counts, as well as in the pH, in early stages of fermentation, samples were collected every 8 hr followed by every 4 hr in middle and later stages. As shown in Figure 2 and Table 1, total LAB number significantly increased from 0 hr (6.22 log cfu/ml) to 32 hr (9.57 log cfu/ml) to 44 hr (9.86 log cfu/ml) to 56 hr (10.83 log cfu/ml) to 80 hr (11.98 log cfu/ml) to 92 hr (12.29 log cfu/ml). Total yeast number rapidly increased after 24 hr (6.80 log cfu/ml), then gradually decreased until 96 hr (4.56 log cfu/ml). After 68 hr of fermentation, we detected the presence of mold (2.7 log cfu/ml), which gradually increased until 96 hr (4.48 log cfu/ml) (Figure 2). We further observed a decrease in the pH from 6.85 at the beginning of fermentation, to 4.66 at 96 hr. After 88 hr of fermentation, we observed floating sour cream (pH 4.82). We collected the superficial sour cream (Z1, Z2, and Z3) as well as underlying curds at 88 hr, 92 hr, and 96 hr, respectively. The average number of viable LAB, yeast, and mold in the Zuohe was 12.18 ± 0.50 log cfu/ml, 6.05 ± 0.30 log cfu/ml and 5.41 ± 0.24 log cfu/ml, respectively (Figure 2 and Table 1). We did not observe differences in the LAB count nor in the temperature between the underlying curds and superficial Zuohe (*p* > .05), whereas the number of viable yeast and mold in the curds was significantly lower than in the Zuohe (*p* < .01 and .05, respectively). Concerning the pH, we observed a significantly higher value in the curds compared with Zuohe (*p* < .05). We hypothesize the lower pH results from the exposure of the superficial sour cream to environmental fungi, as well as to the increased yeast number.

**TABLE 1:** Microbial changes during natural fermentation of milk

Time	LAB	Yeast	Mold	pH	Temperature
0 hr	6.22	4.16	0.00	6.85	6.30
8 hr	6.24	4.15	0.00	6.77	12.00
16 hr	6.11	4.18	0.00	6.74	13.10
24 hr	7.39	6.80	0.00	6.64	13.20
32 hr	9.57	5.26	0.00	6.65	11.50
36 hr	7.65	5.49	0.00	6.60	10.90
40 hr	9.69	4.80	0.00	6.51	11.10
44 hr	9.86	5.73	0.00	6.48	11.40
48 hr	8.76	5.75	0.00	6.34	11.80
52 hr	8.06	6.26	0.00	6.20	12.10
56 hr	10.83	5.77	0.00	6.05	13.10
60 hr	9.56	6.09	0.00	5.89	13.50
64 hr	9.62	5.67	0.00	5.71	13.60
68 hr	9.78	5.29	2.70	5.54	13.70
72 hr	11.82	5.47	3.18	5.33	14.00
76 hr	11.55	4.43	2.70	5.13	15.00
80 hr	11.98	4.34	3.35	5.00	15.80
84 hr	10.10	5.00	3.90	4.85	16.30
88 hr	9.81	3.81	3.40	4.82	16.40
92 hr	12.29	4.08	3.74	4.77	16.30
96 hr	12.05	4.56	4.48	4.66	16.80
Z1	11.61	5.70	5.15	4.67	16.50
Z2	12.57	6.18	5.45	4.55	16.20
Z3	12.37	6.26	5.63	4.53	16.70

The LAB counts in the traditional fermented dairy products from Mongolia, Chigee (naturally fermented mare's milk) from Inner Mongolia, the traditional Bangladeshi fermented milk products (dahi), and kefir grains varied from 3.41 to 9.03 log cfu/ml (Yu et al., 2011), 5.32 to 8.56 log cfu/ml (Guo, Xu, et al., 2019), 6.6 to 8.4 log cfu/g (Nahidul‐Islam et al., 2018), and 4.81 to 8.93 log cfu/g (Witthuhn et al., 2004), respectively. Overall, LAB counts in our fermented samples (the curds: 11.38 ± 1.37 log cfu/ml; Zuohe: 12.18 ± 0.50 log cfu/ml) were higher than in any of the above fermented products tests. In addition, the viable yeast count ranged from 2.41 to 6.98 log cfu/ml in Chigee (Guo, Xu, et al., 2019), 2.8 to 7.8 log CFU/g in the traditional Bangladeshi fermented milk products (Nahidul‐Islam et al., 2018), and 5.18 to 8.57 log cfu/g in kefir grains (Witthuhn et al., 2004). In contrast, yeast counts in our naturally fermented products (the curds: 4.15 ± 0.38 cfu/ml; Zuohe: 6.05 ± 0.30 cfu/ml) were generally lower. We reasoned that the large‐scale, low temperature, and static natural fermentation could contribute to LAB proliferation, which in turn might inhibit the yeast growth.

LAB and yeast often coexist in the process of natural fermentation, such as traditional dairy products (Akabanda et al., 2013; Guo, Xu, et al., 2019; Guo, Ya, et al., 2019; Liu et al., 2015; Nahidul‐Islam et al., 2018; Sun et al., 2014; Witthuhn et al., 2004; Yamei et al., 2019), sourdoughs (Lhomme et al., 2015), traditional Chinese liquor (Li et al., 2013), traditional Korean alcoholic beverages (Jung et al., 2012), Chinese horse bean‐chili‐paste (Lu et al., 2020), fermented Pu‐erh tea (Zhang et al., 2016), and Chinese Sichuan Paocai (Xiao et al., 2018). Although natural fermentation of milk, predominantly resulting from lactic fermentation, the invariable co‐occurrence of LAB and yeast suggests that an interplay between these two might occur, which contributes to the characteristics of dairy products. Yeast play an important role in the production of carbon dioxide and ethanol in naturally fermented dairy products, such as kefir and koumiss. However, the interaction between LAB and yeast has not been extensively studied. Such limitation can be overcome by the study of distinct strains occurring in naturally fermented products (Carbonetto et al., 2020; Mendoza et al., 2010; Sieuwerts et al., 2018; Sudun et al., 2013). The stimulation or inhibition of growth of one, or both, of the cocultured strains does not totally explain the potential interactions between LAB and yeast community in natural fermentation. In this study, despite the gradual increase in LAB as a whole, the dynamics of this process included four significant rise and fall stages (L1, L2, L3, and L4) (Figure 2). More interestingly, the corresponding stages of yeast (Y1, Y2, Y3, and Y4) mirrored the variation of LAB growth, which were similar to the distinct phases of mold growth (M4) (Figure 2). The above results indicate that the fermentative microbiota exhibited antagonist behavior, when comparing bacteria (LAB) to fungi (yeast and mold). We hypothesize that LAB and yeast in coculture may compete for nutrients or that they produce some metabolic substances that inhibit each other's growth.

### 3.2 Dynamic change of bacterial community during consecutive natural fermentation of milk

After removing low‐quality and chimera reads, a total of 2,133,019 bacterial reads (Average ± *SD*: 88,876 ± 4,210) were obtained, and the OTUs of 0 hr to Z3 are shown in Table 2. Chao1, Shannon, Simpson, and Good's coverage were utilized to evaluate bacterial community enrichment and diversity, and shown in Table 2. These indexes demonstrated that the bacterial community was adequately represented, and changes of OTUs and Shannon indicated that bacterial diversity declined during natural fermentation. In addition, there were no significant differences in bacterial diversity between the curds (88 hr, 92 hr, and 96 hr) and Zuohe (Z1, Z2, and Z3) (*p* > .05).

**TABLE 2:** Bacterial diversity indices of 16S rRNA sequencing of the samples from the continuous fermentation process

Time	Reads	OTUs	Chao	Shannon	Simpson	Good's coverage
0 hr	92,188	876	1,150	6.08	0.96	0.9972
8 hr	92,327	873	1,241	4.57	0.89	0.9965
16 hr	88,513	830	1,193	4.12	0.86	0.9961
24 hr	86,996	701	1,080	3.97	0.87	0.9966
32 hr	85,005	685	1,042	4.23	0.89	0.9967
36 hr	81,765	570	1,169	3.97	0.89	0.9961
40 hr	83,742	625	1,100	3.98	0.89	0.9962
44 hr	89,021	758	1,151	4.09	0.88	0.9963
48 hr	86,974	605	1,147	3.93	0.88	0.9961
52 hr	86,837	611	1,016	4.02	0.88	0.9968
56 hr	87,323	526	967	3.52	0.81	0.9968
60 hr	88,950	662	1,001	3.51	0.81	0.9968
64 hr	95,528	610	1,112	3.52	0.80	0.9967
68 hr	94,235	651	1,161	3.58	0.81	0.9968
72 hr	83,684	2,905	2,941	7.57	0.96	0.9968
76 hr	97,999	667	1,209	3.34	0.78	0.9966
80 hr	94,440	855	1,312	4.72	0.88	0.9968
84 hr	88,560	643	1,033	3.99	0.85	0.9969
88 hr	87,692	589	940	3.31	0.77	0.9970
92 hr	93,675	517	883	3.48	0.82	0.9974
96 hr	86,015	572	916	3.61	0.84	0.9972
Z1	85,607	650	992	4.93	0.92	0.9973
Z2	91,116	565	873	4.28	0.90	0.9973
Z3	84,827	515	793	4.05	0.89	0.9973

**FIGURE 3:**
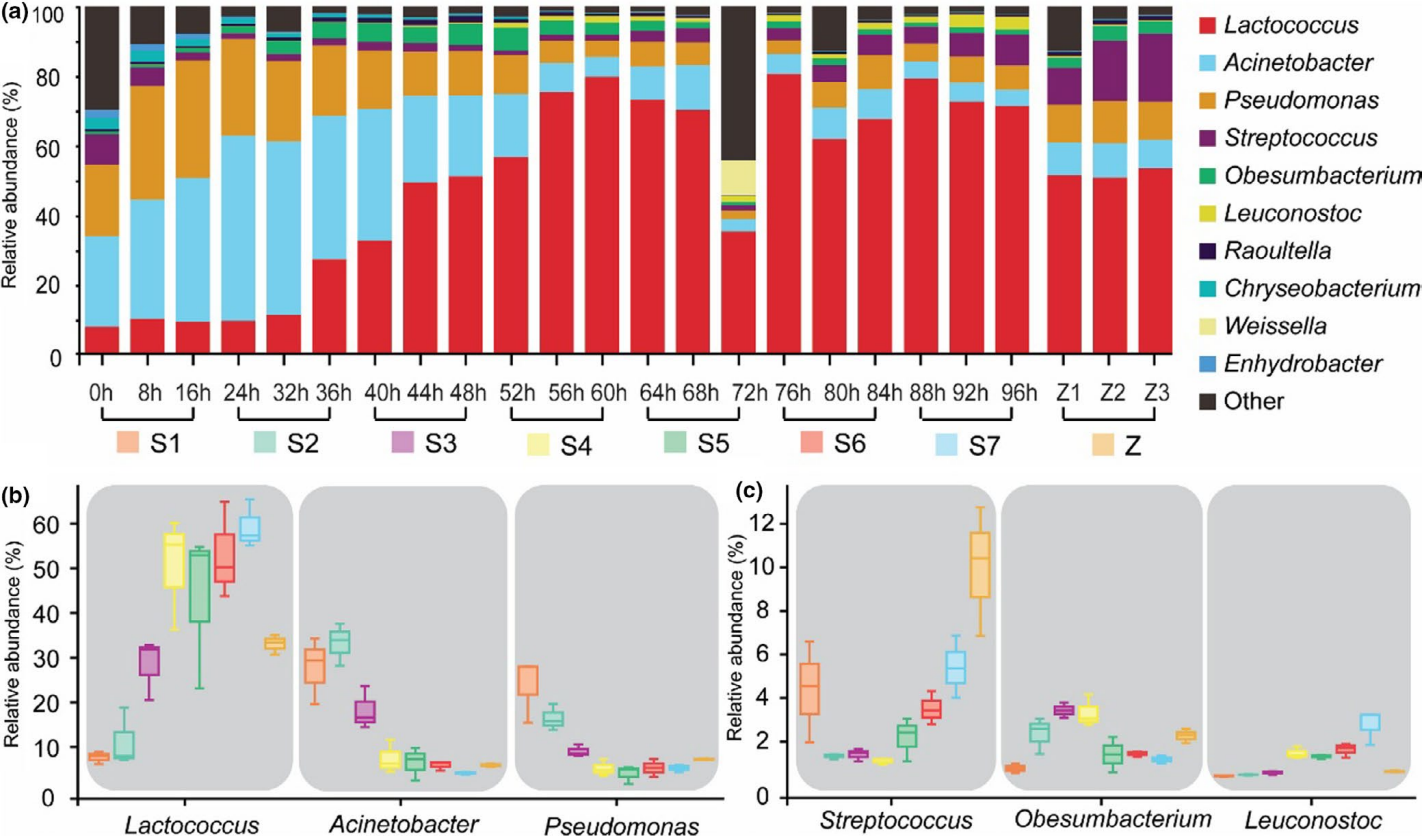
Relative abundance of bacterial sequences (genus level) in the samples from the continuous fermentation process (a). The representative genera change during the continuous three stages of fermentation (b and c). The three consecutive sampling time points can be classified as a fermentative stage, namely as S1 to S7, and the superficial sour cream (Z1, Z2, and Z3) were collected at 88 hr, 92 hr, and 96 hr

The three consecutive sampling time points can be classified as a fermentative stage, namely as S1 to S7 (Figure 3). For the abundance of diverse bacterial phylum group, *Proteobacteria*, *Firmicutes*, *Bacteroidetes,* and *Actinobacteria* represented 69.49 ± 15.37%, 17.42 ± 5.70%, 5.71 ± 3.33% and 2.72 ± 0.69%, respectively in the S1. Finally, *Firmicutes* increased to 67.88 ± 3.83% (S7), whereas *Proteobacteria*, *Bacteroidetes,* and *Actinobacteria* declined to 31.37 ± 3.91% (S7), 0.40 ± 0.17% (S7), and 0.08 ± 0.02% (S7), respectively. In the Zuohe, the abundances of *Proteobacteria*, *Firmicutes*, *Bacteroidetes,* and *Actinobacteria* were 51.49 ± 3.96%, 44.71 ± 3.54%, 0.75 ± 0.67%, and 0.52 ± 0.62%, respectively. Concerning the bacterial genus group (Figure 3a), *Lactococcus*, *Acinetobacter*, *Pseudomonas*, *Streptococcus*, *Obesumbacterium,* and *Leuconostoc* represented 7.56 ± 1.40%, 27.57 ± 7.50%, 23.66 ± 7.24%, 4.34 ± 2.32%, 0.74 ± 0.22%, and 0.06 ± 0.04%, respectively, in S1. In S7, *Lactococcus* and *Leuconostoc* increased to 59.17 ± 5.44% and 2.41 ± 0.80%, respectively (Figure 3b and c), and *Acinetobacter* and *Pseudomonas* declined to 4.00 ± 0.34% and 5.16 ± 0.80%, respectively (Figure 3b). The abundance of *Streptococcus* first decreased to 1.30 ± 0.13% (S2), increasing to 5.38 ± 1.43% during S7 (Figure 3c). In contrast, *Obesumbacterium* increased to 3.40 ± 0.35% during S3, then decreasing to 1.15 ± 0.18% at S7 (Figure 3c). In the Zuohe, the abundances of *Lactococcus*, *Acinetobacter*, *Pseudomonas*, *Streptococcus*, *Obesumbacterium,* and *Leuconostoc* were of 32.91 ± 2.20%, 5.80 ± 0.38%, 7.12 ± 0.18%, 9.98 ± 2.97%, 2.24 ± 0.33%, and 0.27 ± 0.04%, respectively (Figure 3a).

### 3.3 Bacterial community variations in the representative fermentation stage

To assess the bacterial dynamics during natural fermentation, some critical time points (0 hr, 36 hr, 44 hr, 52 hr, 68 hr, 80 hr, 88 hr, 96 hr, Z1 and Z3) were chosen to carry out three biological replicates. A total of 2,667,601 bacterial reads (Average ± *SD*: 88,920 ± 4,110) were obtained, and the OTUs are shown in Table 3. Chao1, Shannon, Simpson, and Good's coverage were used to evaluate bacterial community enrichment and diversity, as shown in Table 3. The alpha indexes demonstrated that the bacterial diversity declined during natural fermentation (*p* < .01), with higher bacterial diversity in the Zuohe than in the final curds (96 hr, *p* < .01).

**TABLE 3:** Bacterial diversity indices of 16S rRNA sequencing in the representative samples of fermentation

Time	Reads	OTUs	Chao	Shannon	Simpson	Good's coverage
0 hr	91,009 ± 2,163	876 ± 23	1,230 ± 31	5.06 ± 0.95	0.908 ± 0.044	0.9965 ± 0.0005
36 hr	88,032 ± 5,806	675 ± 38	1,175 ± 12	4.17 ± 0.04	0.902 ± 0.001	0.9961 ± 0.0003
44 hr	86,802 ± 3,976	794 ± 86	1,187 ± 84	4.14 ± 0.01	0.884 ± 0.012	0.9960 ± 0.0001
52 hr	85,756 ± 4,636	739 ± 83	1,113 ± 51	4.25 ± 0.38	0.886 ± 0.017	0.9964 ± 0.0002
68 hr	90,167 ± 4,471	685 ± 17	1,182 ± 62	3.32 ± 0.27	0.781 ± 0.027	0.9963 ± 0.0002
80 hr	91,884 ± 2,288	762 ± 138	1,229 ± 168	4.20 ± 0.55	0.859 ± 0.027	0.9966 ± 0.0002
88 hr	92,473 ± 4,263	684 ± 91	1,099 ± 99	3.62 ± 0.38	0.795 ± 0.021	0.9968 ± 0.0003
96 hr	86,276 ± 1,976	669 ± 100	1,037 ± 107	3.62 ± 0.03	0.834 ± 0.001	0.9966 ± 0.0003
Z1	90,565 ± 5,338	703 ± 36	1,136 ± 106	4.48 ± 0.44	0.901 ± 0.013	0.9966 ± 0.0006
Z3	86,235 ± 2,042	599 ± 19	999 ± 70	4.04 ± 0.07	0.890 ± 0.006	0.9968 ± 0.0002

**FIGURE 4:**
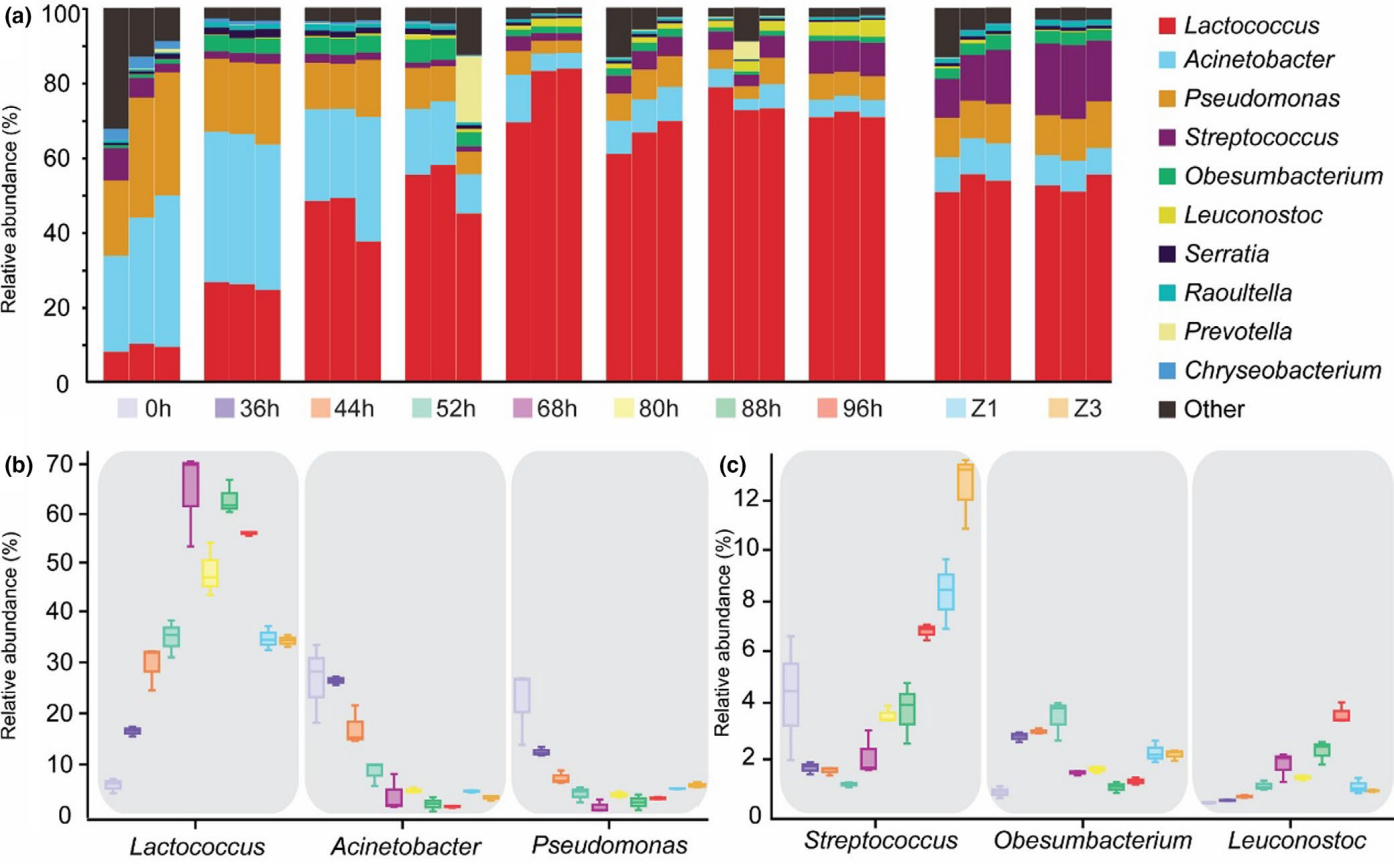
Relative abundance of bacterial sequences (genus level) in the representative samples of fermentation (a). The representative genera change during the representative stages of fermentation (b and c)

For bacterial phylum group, the abundance of *Firmicutes* (*p* < .01) increased during natural fermentation, whereas *Proteobacteria* (*p* < .01), *Bacteroidetes* (*p* < .05), and *Actinobacteria* (*p* < .05) declined from 0 hr to 96 hr (Table 4). In addition, the Zuohe exhibited higher abundance of *Proteobacteria* than the curds, and less *Firmicutes* (*p* < .01) (Table 4). The results are in line with previous investigations, showing the four major bacterial phyla present in traditionally fermented dairy products (Gesudu et al., 2016; Guo, Ya, et al., 2019; Sun et al., 2014; Yamei et al., 2019). Moreover, although we observed a predominance of the *Proteobacteria* in early fermentation stages, its abundance decreased concomitantly with the increase in *Firmicutes*, as described previously in traditionally fermented foods, such as in traditional Korean salted seafood (Lee et al., 2014), traditional Chinese fish sauce (Du et al., 2019), and traditional Indian food idli (Mandhania et al., 2019). Concerning bacterial genus group (Figure 4 and Table 4), *Lactococcus* and *Leuconostoc* increased significantly (*p* < .01), whereas *Acinetobacter* and *Pseudomonas* declined significantly (*p* < .01). *Streptococcus* increase, after an initial decrease in abundance (Figure 4c and Table 4) (*p* < .01), whereas *Obesumbacterium* initially increased, followed by a significant decrease (Figure 4c and Table 4) (*p* < .01). The abundances of *Lactococcus* and *Leuconostoc* were lower in the Zuohe than in the curds (*p* < .01). In contrast, *Acinetobacter*, *Pseudomonas*, *Streptococcus,* and *Obesumbacterium* were more present in the Zuohe than in the curds (*p* < .05) (Table 4). Regarding the bacterial species group, *Lactococcus lactis* showed a significant increase (*p* < .01), contrary to *Acinetobacter johnsonii*, which abundance declined significantly (*p* < .01) (Table 4). Furthermore, the amount of *Lactococcus lactis* in the Zuohe was lower than in the curds (*p* < .01), whereas *Acinetobacter johnsonii* was higher (*p* < .05) (Table 4).

**TABLE 4:** The abundance (%) of diverse bacterial phylum, genus, and species during natural fermentation of milk

	0 hr	36 hr	44 hr	52 hr	68 hr	80 hr	88 hr	96 hr	Z1	Z3
*Proteobacteria*	69.44 ± 15.39	78.48 ± 0.70	65.27 ± 4.64	52.82 ± 9.81	31.56 ± 8.63	38.92 ± 3.00	24.93 ± 8.05	32.96 ± 0.62	49.79 ± 2.50	50.86 ± 0.78
*Firmicutes*	17.50 ± 5.81	20.14 ± 0.68	33.37 ± 4.52	40.69 ± 1.48	67.54 ± 8.74	55.29 ± 3.66	72.19 ± 4.90	66.16 ± 0.75	45.93 ± 2.27	48.19 ± 0.70
*Bacteroidetes*	5.65 ± 3.23	0.88 ± 0.17	0.77 ± 0.03	5.59 ± 8.51	0.31 ± 0.10	1.49 ± 1.37	2.30 ± 3.11	0.42 ± 0.01	1.13 ± 0.59	0.32 ± 0.06
*Actinobacteria*	2.73 ± 0.70	0.33 ± 0.08	0.26 ± 0.04	0.37 ± 0.33	0.16 ± 0.09	0.24 ± 0.22	0.12 ± 0.09	0.10 ± 0.02	0.63 ± 0.55	0.11 ± 0.01
*Lactococcus*	7.57 ± 1.37	17.85 ± 0.91	30.55 ± 4.22	35.71 ± 3.54	63.51 ± 9.07	48.25 ± 5.07	62.03 ± 3.12	55.56 ± 0.39	35.53 ± 2.27	35.07 ± 1.12
*Acinetobacter*	27.67 ± 7.53	27.52 ± 0.78	18.49 ± 3.72	10.14 ± 2.34	5.66 ± 3.52	6.56 ± 0.46	3.97 ± 1.32	3.51 ± 0.20	6.45 ± 0.25	5.19 ± 0.46
*Pseudomonas*	23.63 ± 7.27	13.93 ± 0.81	8.96 ± 1.28	5.94 ± 1.48	3.49 ± 1.19	5.75 ± 0.57	4.30 ± 1.47	5.10 ± 0.21	6.91 ± 0.07	7.58 ± 0.50
*Streptococcus*	4.35 ± 2.33	1.65 ± 0.23	1.54 ± 0.15	1.02 ± 0.10	2.09 ± 0.84	3.62 ± 0.31	3.80 ± 1.15	6.78 ± 0.30	8.23 ± 1.31	12.21 ± 1.38
*Obesumbacterium*	0.73 ± 0.22	2.82 ± 0.19	3.02 ± 0.10	3.54 ± 0.76	1.46 ± 0.09	1.59 ± 0.13	0.92 ± 0.21	1.14 ± 0.13	2.22 ± 0.41	2.15 ± 0.20
*Leuconostoc*	0.06 ± 0.04	0.15 ± 0.02	0.29 ± 0.05	0.70 ± 0.18	1.48 ± 0.55	1.00 ± 0.09	2.00 ± 0.45	3.11 ± 0.39	0.39 ± 0.29	0.23 ± 0.04
*Lactococcus lactis*	4.92 ± 0.95	13.51 ± 0.65	24.29 ± 4.39	24.26 ± 2.58	37.60 ± 0.83	30.52 ± 3.07	37.87 ± 2.60	27.26 ± 0.49	18.83 ± 0.73	18.73 ± 1.05
*Acinetobacter johnsonii*	16.24 ± 5.94	16.22 ± 0.36	10.19 ± 2.49	5.31 ± 1.33	3.70 ± 2.63	4.31 ± 0.31	2.53 ± 0.78	2.17 ± 0.10	3.95 ± 0.10	3.00 ± 0.18

Each of the previously mentioned bacteria as a key role during natural fermentation. *Lactococcus* strains are widely used for industrial production of fermented dairy products. Besides the capacity to extend the shelf‐life of dairy products, increasing amounts of *Lactococcus* during milk natural fermentation are at the basis of the sour and fermentative fragrances (Casalta & Montel, 2008; Cavanagh et al., 2015; Song et al., 2017). *Leuconostoc* spp. are a major contributor to the production of aromatic compounds during dairy fermentations (Endo et al., 2020). *Streptococcus thermophilus* is a species of lactic acid bacteria which is essential for the manufacturing of many types of fermented dairy products (Harnett et al., 2020). *Acinetobacter* (Kämpfer, 2014), *Pseudomonas* (Dodd, 2014), and *Obesumbacterium* (Enterobacteriaceae family) (Patel et al., 2014) are regarded as spoilage microbes for food, bringing about concerns human health.

**FIGURE 7:**
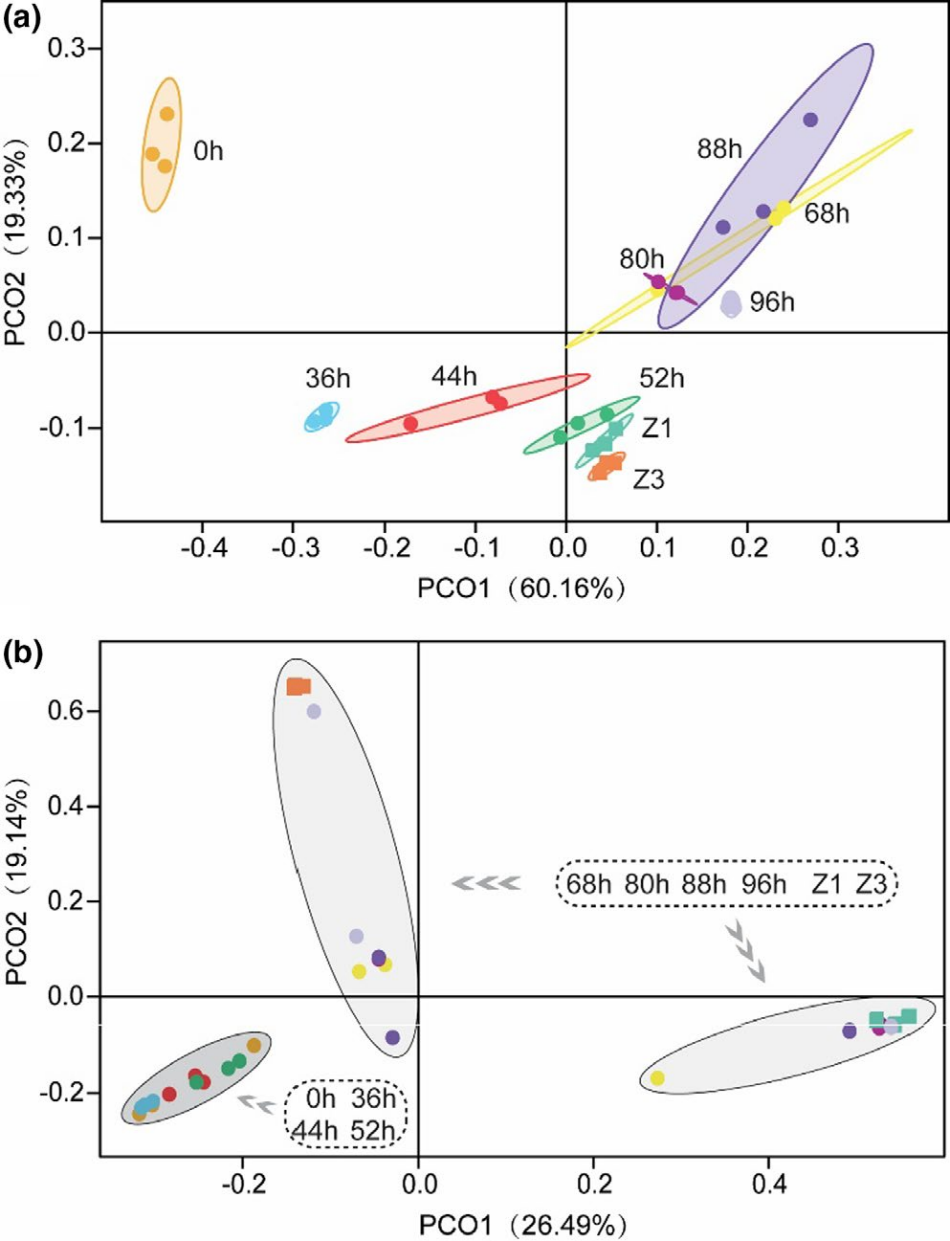
UniFrac principal coordinate analyses of bacterial (a) and fungal (b) diversity in the representative samples of fermentation

Multivariate analysis was performed to compare the bacterial community structures from naturally fermented samples. As demonstrated in Figure 7a, PcoA, which uses species‐level OTUs, showed significant differences among samples from different fermentation time points (ANOSIM, *R* = 0.82, *p* = .001), supporting the successional dynamics of bacteria. In addition, samples from different fermentation time points were largely separated in the bray analysis (accounting for 60.16% and 19.33% of the total variance by the two principal components, respectively). In conclusion, the process of natural fermentation is accompanied by the growth of viable LAB count and the decay of bacterial diversity. At the same time, dairy fermentative microorganisms gradually increase, whereas potential spoilage or pathogenic microbes decrease dramatically.

### 3.4 Dynamic change of fungal community during consecutive natural fermentation of milk

After removal of the low‐quality and chimera reads, a total of 1,856,760 fungal reads (Average ± *SD*: 77,365 ± 10,108) were obtained. OTUs of 0 hr to Z3 are shown in Table 5. Chao1, Shannon, Simpson, and Good's coverage were used to evaluate fungal community enrichment and diversity, as shown in Table 5. The indexes demonstrated that the fungal community was adequately represented, with changes in OTUs and Shannon indicating that the fungal diversity declined during natural fermentation. In addition, there were no significant differences between the fungal diversity of the curds (88 hr, 92 hr, and 96 hr) and Zuohe (Z1, Z2, and Z3) (*p* > .05).

**TABLE 5:** Fungal diversity indices of ITS sequencing of the samples from the continuous fermentation process

Time	Reads	OTUs	Chao	Shannon	Simpson	Good's coverage
0 hr	77,641	231	194	4.31	0.91	0.9992
8 hr	86,093	320	289	5.64	0.96	0.9996
16 hr	68,454	240	246	4.56	0.93	0.9985
24 hr	53,424	243	220	4.07	0.88	0.9983
32 hr	78,491	250	230	3.91	0.88	0.9991
36 hr	81,583	263	223	3.71	0.83	0.9987
40 hr	72,463	243	179	4.39	0.91	0.9993
44 hr	56,190	249	214	4.46	0.93	0.9990
48 hr	71,502	246	202	4.39	0.93	0.9992
52 hr	91,306	260	206	5.46	0.96	0.9996
56 hr	74,516	213	135	2.77	0.58	0.9993
60 hr	68,181	208	157	3.18	0.66	0.9981
64 hr	75,622	214	167	1.77	0.38	0.9986
68 hr	81,639	198	118	1.49	0.31	0.9995
72 hr	73,423	265	209	1.42	0.28	0.9984
76 hr	73,777	230	162	2.43	0.52	0.9989
80 hr	77,634	229	133	1.32	0.27	0.9993
84 hr	83,745	213	124	1.37	0.28	0.9994
88 hr	92,600	359	230	3.82	0.85	0.9993
92 hr	70,286	197	105	2.62	0.57	0.9991
96 hr	90,660	175	117	1.06	0.22	0.9997
Z1	83,372	127	103	0.43	0.10	0.9998
Z2	82,948	97	96	0.80	0.27	0.9997
Z3	91,210	60	64	0.20	0.04	0.9998

**FIGURE 5:**
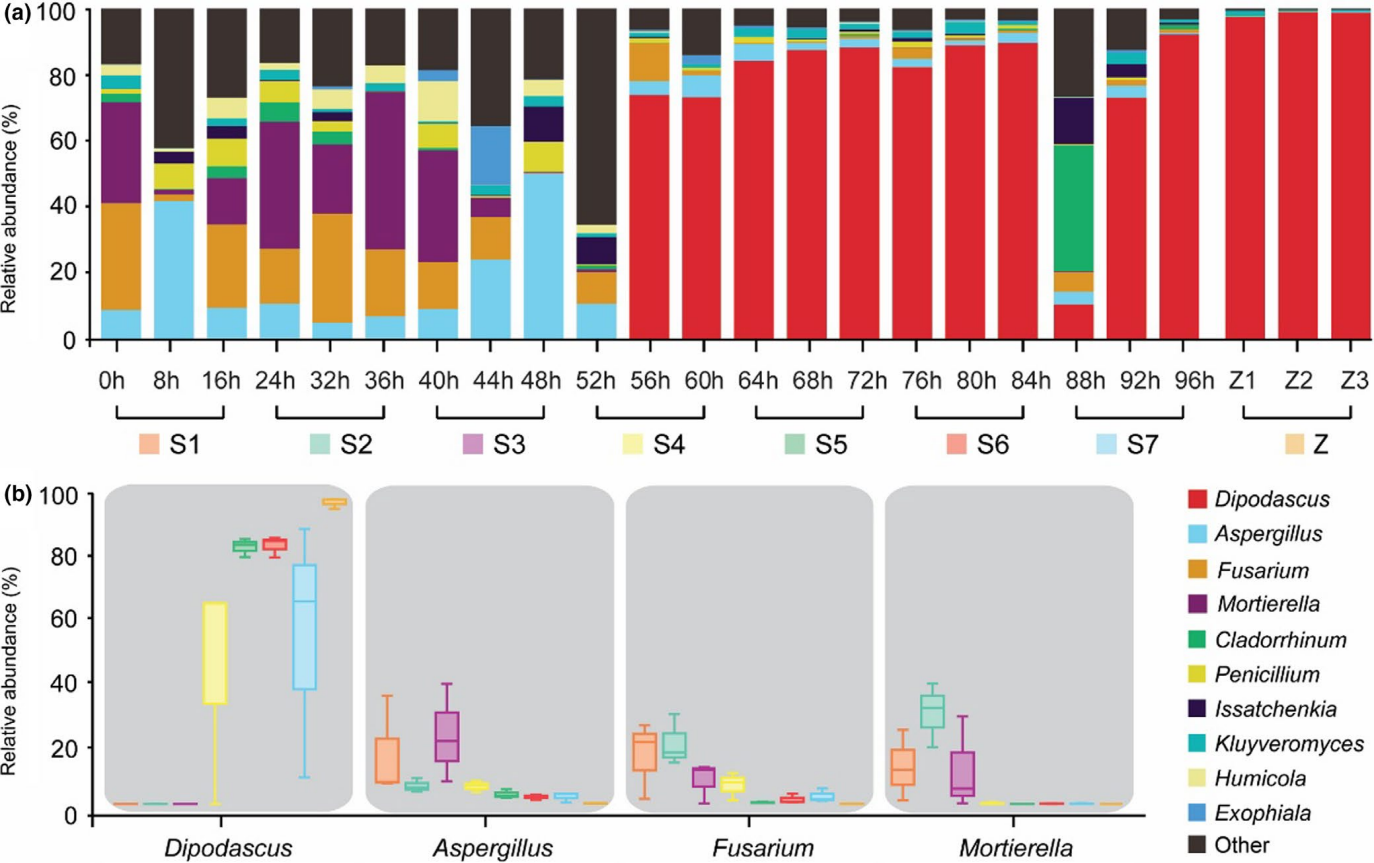
Relative abundance of fungal sequences (genus level) in the samples from the continuous fermentation process (a). The representative genera change during the continuous three stages of fermentation (b). The three consecutive sampling time points can be classified as a fermentative stage, namely as S1 to S7, and the superficial sour cream (Z1, Z2, and Z3) were collected at 88 hr, 92 hr, and 96 hr

The fungal sequencing reads were classified at the phylum and genus levels. At the phylum level, the phyla *Ascomycota*, *Mortierellomycota,* and *Basidiomycota* were detected at 81.78 ± 12.17%, 12.02 ± 11.35%, and 3.46 ± 2.87%, respectively, during S1. Finally, during S7, *Ascomycota* increased to 96.55 ± 0.67%, whereas *Mortierellomycota* and *Basidiomycota* declined to 0.08 ± 0.14% and 0.96 ± 0.94%, respectively. In the Zuohe, *Ascomycota*, *Mortierellomycota,* and *Basidiomycota* represented 98.40 ± 0.86%, 0, and 0.30 ± 0.10% of fungi community, respectively. Concerning the fungal genus group (Figure 5a), *Dipodascus*, *Aspergillus*, *Fusarium,* and *Mortierella* were detected at 0.01 ± 0.02%, 16.19 ± 16.13%, 15.65 ± 12.41%, and 12.02 ± 11.35%, respectively, during S1. At S7, *Dipodascus* increased to 54.03 ± 41.09% (Figure 5b), whereas *Aspergillus*, *Fusarium,* and *Mortierella* declined to 2.34 ± 1.60%, 2.51 ± 2.17%, and 0.08 ± 0.14%, respectively (Figure 5b). In the Zuohe, *Dipodascus*, *Aspergillus*, *Fusarium,* and *Mortierella* represented 96.90 ± 1.69%, 0.19 ± 0.11%, 0.04 ± 0.05%, and 0, respectively (Figure 5a).

### 3.5 Fungal community variations in the representative fermentation stage

**FIGURE 6:**
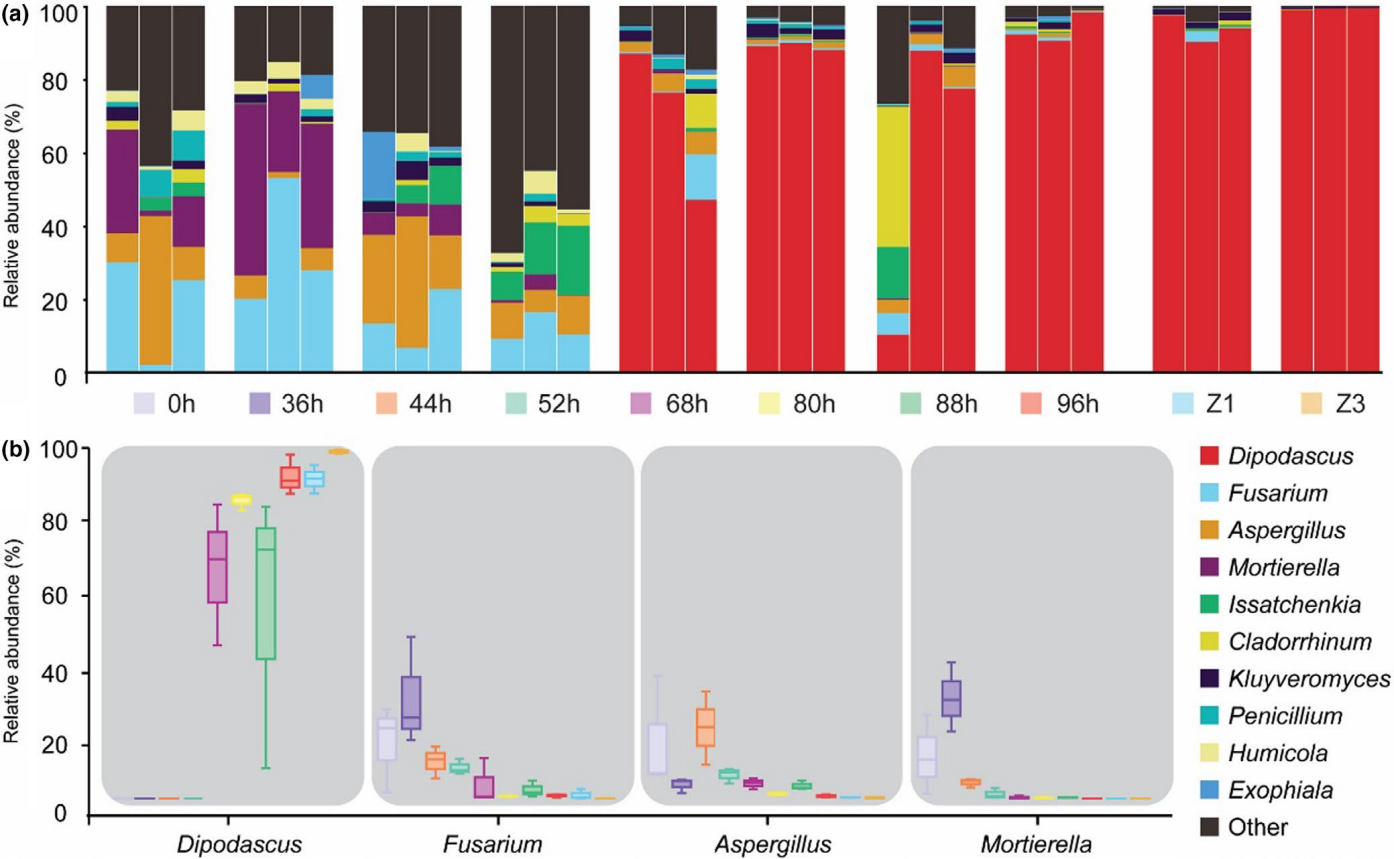
Relative abundance of fungal sequences (genus level) in the representative samples of fermentation (a). The representative genera change during the representative stages of fermentation (b)

To assess fungal dynamics during natural fermentation, key fermentation time points (0 hr, 36 hr, 44 hr, 52 hr, 68 hr, 80 hr, 88 hr, 96 hr, Z1, and Z3) were chosen to carry out three biological replicates. A total of 2,386,130 fungal reads (Average ± *SD*: 79,538 ± 9,264) were obtained. OTUs are shown in Table 6. Chao1, Shannon, Simpson, and Good's coverage were used to evaluate fungal community enrichment and diversity, as shown in Table 6. The alpha indexes showed that fungal diversity declined during natural fermentation (*p* < .01), with no significant differences in the fungal diversity between the curds (96 hr) and Zuohe (Z3) (*p* > .05). At the phylum level, the phyla *Ascomycota* (*p* < .01) increased during natural fermentation, whereas *Mortierellomycota* (*p* < .01) and *Basidiomycota* (*p* < .01) declined from 0 hr to 96 hr (Table 7). At the genus level (Figure 6 and Table 7), *Dipodascus* increased significantly (*p* < .01), and *Aspergillus*, *Fusarium,* and *Mortierella* showed an initial increased (0 hr to 44 hr) followed by a marked reduction (Figure 6b and Table 7) (*p* < .01). Concerning fungal species group, *Dipodascus australiensis* increased significantly (*p* < .01), which contrasts with *Fusarium solani*, which declined after an initial increase (from 0 hr to 36 hr, *p* < .01) (Table 7). We did not observe significant differences in terms of the abundances of fungal phylum, genus, and species between the curds and Zuohe (Table 7) (*p* > .05).

**TABLE 6:** Fungal diversity indices of ITS sequencing in the representative samples of fermentation

Time	Reads	OTUs	Chao	Shannon	Simpson	Good's coverage
0 hr	77,396 ± 8,822	272 ± 51	240 ± 43	4.86 ± 0.72	0.93 ± 0.03	0.9991 ± 0.0005
36 hr	72,668 ± 9,947	259 ± 8	232 ± 7	3.60 ± 0.37	0.81 ± 0.06	0.9987 ± 0.0002
44 hr	67,488 ± 12,306	297 ± 38	250 ± 42	4.80 ± 0.31	0.94 ± 0.01	0.9988 ± 0.0001
52 hr	85,059 ± 6,122	272 ± 12	211 ± 7	5.31 ± 0.23	0.959 ± 0.003	0.9994 ± 0.0002
68 hr	72,919 ± 9,129	231 ± 36	155 ± 23	2.75 ± 0.96	0.59 ± 0.19	0.9985 ± 0.0008
80 hr	80,976 ± 3,847	196 ± 23	156 ± 21	1.43 ± 0.13	0.29 ± 0.03	0.9992 ± 0.0001
88 hr	82,370 ± 9,352	285 ± 96	183 ± 49	2.56 ± 1.18	0.56 ± 0.27	0.9990 ± 0.0004
96 hr	84,656 ± 8,965	172 ± 56	133 ± 50	0.90 ± 0.58	0.18 ± 0.13	0.9994 ± 0.0005
Z1	83,491 ± 1,800	165 ± 28	149 ± 40	0.98 ± 0.59	0.22 ± 0.15	0.9996 ± 0.0002
Z3	88,353 ± 2,866	71 ± 8	77 ± 4	0.17 ± 0.08	0.03 ± 0.02	0.99977 ± 0.00002

**TABLE 7:** The abundance (%) of diverse fungal phylum, genus, and species changes during natural fermentation of milk

	0 hr	36 hr	44 hr	52 hr	68 hr	80 hr	88 hr	96 hr	Z1	Z3
*Ascomycota*	81.78 ± 12.18	64.22 ± 10.68	85.03 ± 3.30	88.61 ± 1.52	97.87 ± 1.02	97.42 ± 0.38	96.79 ± 1.34	99.37 ± 0.55	97.09 ± 0.52	99.47 ± 0.54
*Mortierellomycota*	12.02 ± 11.36	28.57 ± 9.85	4.47 ± 1.28	1.22 ± 1.48	0.25 ± 0.42	0.07 ± 0.11	0.17 ± 0.07	0	0.01 ± 0.01	0
*Basidiomycota*	3.46 ± 2.88	2.79 ± 1.31	7.97 ± 1.58	6.79 ± 3.91	1.51 ± 0.98	0.67 ± 0.19	0.46 ± 0.07	0.33 ± 0.26	0.74 ± 0.48	0.19 ± 0.11
*Dipodascus*	0.01 ± 0.02	0	0	0	65.08 ± 20.23	84.74 ± 2.46	54.11 ± 39.96	91.65 ± 5.67	90.90 ± 4.08	98.71 ± 0.55
*Fusarium*	15.65 ± 12.41	28.49 ± 5.47	10.48 ± 4.55	8.76 ± 2.21	4.06 ± 6.43	0.56 ± 0.15	2.40 ± 2.34	0.74 ± 0.51	0.97 ± 1.49	0.01 ± 0.01
*Aspergillus*	16.20 ± 16.15	3.85 ± 2.10	20.05 ± 10.44	6.64 ± 2.19	4.23 ± 1.57	1.25 ± 0.35	3.57 ± 1.28	0.62 ± 0.40	0.20 ± 0.05	0.12 ± 0.16
*Mortierella*	12.02 ± 11.36	28.57 ± 9.85	4.47 ± 1.28	1.22 ± 1.48	0.25 ± 0.42	0.07 ± 0.11	0.17 ± 0.07	0	0.01 ± 0.01	0
*Dipodascus australiensis*	0.01 ± 0.02	0	0	0	65.08 ± 20.23	84.74 ± 2.46	54.11 ± 39.96	91.65 ± 5.67	90.90 ± 4.08	98.71 ± 0.55
*Fusarium solani*	14.77 ± 11.48	28.45 ± 15.45	10.44 ± 4.53	8.72 ± 2.19	3.11 ± 5.15	0.36 ± 0.28	1.90 ± 2.21	0.34 ± 0.28	0.40 ± 0.54	0.01 ± 0.01

The yeast *Dipodascus* has been identified in naturally fermented dairy products from Inner Mongolia (Guo, Ya, et al., 2019; Yamei et al., 2019). *Aspergillus*, *Fusarium,* and *Mortierella* present potential threats to cattle and humans that might result from fungal abortion, mycotoxins, and aspergillosis (Davies et al., 2010; Foster, 2017; Rodrigues, 2016; Thrane, 2014). We reasoned that, in the context of dairy products, *Aspergillus*, *Fusarium,* and *Mortierella* behave as spoilage microorganisms. After the initial growth (0 hr to 44 hr), these three genera become feeble and gradually disappear. Raw milk is a favorable environment that supports their initial growth. However, growth is likely inhibited by the increasing amounts of LAB and, consequently, of increased lactic acid concentration. This might inhibit the growth of these fungi in the stages of metaphase and anaphase of natural fermentation. A reduction in potentially pathogenic molds throughout the natural fermentation meant that potential mycotoxins were produced in the natural fermentation of cow's milk. As a result, further studies will be carried out for the detection and quantification of mycotoxins in the traditionally fermented milk.

Multivariate analysis was performed to compare the fungal community structures from samples during natural fermentation. As demonstrated in Figure 7b, PcoA, which uses species‐level OTUs, showed significant differences among two groups of samples from the early stage (0 hr, 36 hr, 44 hr, and 52 hr) of the fermentation and the late (68 hr, 80 hr, 88 hr, 96 hr, Z1, and Z3) (ANOSIM, *R* = 0.6, *p* = .001). Still, the analysis showed overlapping between samples from the two groups. The results supported the successive changes of fungi, *Dipodascus* increased significantly after 52 hr of fermentation, whereas *Aspergillus*, *Fusarium,* and *Mortierella* decreased gradually during the early stage (0 hr, 36 hr, 44 hr, and 52 hr) of the fermentation. In addition, the values of samples from different fermentation time points were largely separated in the bray analysis (accounting for 26.49% and 19.14% of the total variance by the two principal components, respectively).

## 4 CONCLUSIONS

Traditional dairy products derived from milk's natural fermentation are worldwide used for their nutrient content, fermented flavor and long shelf‐life, providing substantial benefits for human health. Still, the presence of potentially pathogenic microorganisms derived from natural fermentation, together with lack of proper sanitary conditions raised public concerns. In this study, we found that the beneficial bacteria and fungi (e.g., *Lactococcus*, *Streptococcus*, *Leuconostoc*, *Dipodascus*) are gradually increased in concentration and that potentially pathogenic microorganisms (e.g., *Acinetobacter*, *Pseudomonas*, *Fusarium*, *Aspergillus*, *Mortierella*) decrease during the process of natural fermentation. The results support the health beneficial properties of naturally fermented products and highlight the nomadic dairy culture for consumption of naturally fermented milk. Natural, static milk fermentation forms curds and Zuohe (traditional sour cream). Although there were no significant differences in the cultured LAB between the underlying curds and superficial Zuohe, the potentially detrimental bacteria (*Acinetobacter*, *Pseudomonas*, *Fusarium*) were significantly increased in the second, which results in an increase of its bacterial diversity. Furthermore, the Zuohe was significantly more enriched in yeast and mold than the curds. However, there were no significant differences in fungi diversity in terms of genus and species between the two types of fermented samples. Given that the Zuohe was at the surface, and thus exposed to environmental microorganisms, it was expected that the potentially detrimental bacteria, yeast, and mold in the air were also significantly more enriched in the Zuohe than in the underlying curds.

## CONFLICT OF INTEREST

All authors declare no conflict of interest.

## AUTHOR CONTRIBUTIONS

**Wei‐Liang Xu:** Data curation‐Equal, Software‐Equal, Validation‐Equal, Writing‐review & editing‐Equal. **Chun‐Dong Li:** Data curation‐Equal, Methodology‐Equal, Software‐Equal, Validation‐Equal. **Yuan‐Sheng Guo:** Data curation‐Equal, Software‐Equal, Validation‐Equal. **Yi Zhang:** Software‐Equal, Validation‐Equal. **Mei Ya:** Software‐Equal, Validation‐Equal. **Liang Guo:** Conceptualization‐Lead, Funding acquisition‐Lead, Investigation‐Lead, Project administration‐Lead, Writing‐original draft‐Lead, Writing‐review & editing‐Lead.

## ETHICAL APPROVAL

This study does not involve any human or animal testing.

## Supporting information

Table S1

## References

[fsn32174-bib-0001] Akabanda, F. , Owusu‐Kwarteng, J. , Tano‐Debrah, K. , Glover, R. L. , Nielsen, D. S. , & Jespersen, L. (2013). Taxonomic and molecular characterization of lactic acid bacteria and yeasts in nunu, a Ghanaian fermented milk product. Food Microbiology, 34(2), 277–283. 10.1016/j.fm.2012.09.025 23541194

[fsn32174-bib-0002] Bornaz, S. , Guizani, N. , Sammari, J. , Allouch, W. , Sahli, A. , & Attia, H. (2010). Physicochemical properties of fermented Arabian mares' milk. International Dairy Journal, 20(7), 500–505. 10.1016/j.idairyj.2010.02.001

[fsn32174-bib-0003] Carbonetto, B. , Nidelet, T. , Guezenec, S. , Perez, M. , Segond, D. , & Sicard, D. (2020). Interactions between *Kazachstania humilis* yeast species and lactic acid bacteria in sourdough. Microorganisms, 8(2), 240. 10.3390/microorganisms8020240 PMC7074792 32053958

[fsn32174-bib-0004] Casalta, E. , & Montel, M. C. (2008). Safety assessment of dairy microorganisms: The *Lactococcus* genus. International Journal of Food Microbiology, 126(3), 271–273. 10.1016/j.ijfoodmicro.2007.08.013 17976847

[fsn32174-bib-0005] Cavanagh, D. , Fitzgerald, G. F. , & McAuliffe, O. (2015). From field to fermentation: The origins of *Lactococcus lactis* and its domestication to the dairy environment. Food Microbiology, 47, 45–61. 10.1016/j.fm.2014.11.001 25583337

[fsn32174-bib-0006] China National Food Safety Standard . (2016a). GB 4789.35‐2016. Detection of food microorganisms ‐ lactic acid bacteria. Ministry of Health of the People's Republic of China, National Food Safety Standard.

[fsn32174-bib-0007] China National Food Safety Standard (2016b). GB 4789.15‐2016. Detection of food microorganisms ‐ yeast and mold. Ministry of Health of the People's Republic of China, National Food Safety Standard.

[fsn32174-bib-0008] Davies, J. L. , Ngeleka, M. , & Wobeser, G. A. (2010). Systemic infection with *Mortierella wolfii* following abortion in a cow. Canadian Veterinary Journal‐Revue Veterinaire Canadienne, 51(12), 1391–1393. PMC2978994 21358934

[fsn32174-bib-0009] Dodd, C. E. R. (2014). PSEUDOMONAS | Introduction. In C. A. Batt , & M. L. Tortorello (Eds), Encyclopedia of food microbiology (2nd edn, pp. 244–247). Academic Press.

[fsn32174-bib-0010] Du, F. , Zhang, X. , Gu, H. , Song, J. , & Gao, X. (2019). Dynamic changes in the bacterial community during the fermentation of traditional Chinese fish sauce (TCFS) and their correlation with TCFS quality. Microorganisms, 7(9), 371. 10.3390/microorganisms7090371 PMC6780869 31546947

[fsn32174-bib-0011] Edgar, R. C. (2013). UPARSE: Highly accurate OTU sequences from microbial amplicon reads. Nature Methods, 10(10), 996–998. 10.1038/nmeth.2604 23955772

[fsn32174-bib-0012] Endo, A. , Maeno, S. , & Liu, S. Q. (2020). Lactic acid bacteria: *Leuconostoc* spp. reference module in food science. Elsevier.

[fsn32174-bib-0013] Foster, R. A. (2017). Chapter 18 ‐ Female reproductive system and MAMMAE. In J. F. Zachary (Ed.), Pathologic Basis of Veterinary Disease (6th edn, pp. 1147–1193). Mosby.

[fsn32174-bib-0014] Gao, M. L. , Hou, H. M. , Teng, X. X. , Zhu, Y. L. , Hao, H. S. , & Zhang, G. L. (2017). Microbial diversity in raw milk and traditional fermented dairy products (Hurood cheese and Jueke) from Inner Mongolia, China. Genetics and Molecular Research, 16(1). 10.4238/gmr16019451 28290619

[fsn32174-bib-0015] Gesudu, Q. , Zheng, Y. , Xi, X. , Hou, Q. C. , Xu, H. , Huang, W. , Zhang, H. , Menghe, B. , & Liu, W. (2016). Investigating bacterial population structure and dynamics in traditional koumiss from Inner Mongolia using single molecule real‐time sequencing. Journal of Dairy Science, 99(10), 7852–7863. 10.3168/jds.2016-11167 27522429

[fsn32174-bib-0016] Guo, L. , Xu, W. L. , Li, C. D. , Ya, M. , Guo, Y. S. , Qian, J. P. , & Zhu, J. J. (2019). Production technology, nutritional, and microbiological investigation of traditionally fermented mare milk (Chigee) from Xilin Gol in China. Food Science & Nutrition, 8(1), 257–264. 10.1002/fsn3.1298 31993151 PMC6977523

[fsn32174-bib-0017] Guo, L. , Ya, M. , Guo, Y. S. , Xu, W. L. , Li, C. D. , Sun, J. P. , Zhu, J. J. , & Qian, J. P. (2019). Study of bacterial and fungal community structures in traditional koumiss from Inner Mongolia. Journal of Dairy Science, 102(3), 1972–1984. 10.3168/jds.2018-15155 30639001

[fsn32174-bib-0018] Harnett, J. , Patrick, A. , Caddick, C. , Pearce, L. , & Davey, G. (2020). Streptococcus thermophilus. Reference module in food science. Elsevier.

[fsn32174-bib-0019] Jung, M. J. , Nam, Y. D. , Roh, S. W. , & Bae, J. W. (2012). Unexpected convergence of fungal and bacterial communities during fermentation of traditional Korean alcoholic beverages inoculated with various natural starters. Food Microbiology, 30(1), 112–123. 10.1016/j.fm.2011.09.008 22265291

[fsn32174-bib-0020] Kämpfer, P. (2014). Acinetobacter. In C. A. Batt , & M. L. Tortorello (Ed.), Encyclopedia of food microbiology (2nd edn, pp. 11–17). Academic Press.

[fsn32174-bib-0021] Karami, S. , Roayaei, M. , Hamzavi, H. , Bahmani, M. , Hassanzad‐Azar, H. , Leila, M. , & Rafieian‐Kopaei, M. (2017). Isolation and identification of probiotic *Lactobacillus* from local dairy and evaluating their antagonistic effect on pathogens. International Journal of Pharmaceutical Investigation, 7(3), 137–141. 10.4103/jphi.JPHI_8_17 29184826 PMC5680649

[fsn32174-bib-0022] Koljalg, U. , Larsson, K. H. , Abarenkov, K. , Nilsson, R. H. , Alexander, I. J. , Eberhardt, U. , Erland, S. , Hoiland, K. , Kjoller, R. , Larsson, E. , Pennanen, T. , Sen, R. , Taylor, A. F. , Tedersoo, L. , Vralstad, T. , & Ursing, B. M. (2005). UNITE: A database providing web‐based methods for the molecular identification of ectomycorrhizal fungi. New Phytologist, 166(3), 1063–1068. 10.1111/j.1469-8137.2005.01376.x 15869663

[fsn32174-bib-0023] Lee, S. H. , Jung, J. Y. , & Jeon, C. O. (2014). Microbial succession and metabolite changes during fermentation of saeu‐jeot: Traditional Korean salted seafood. Food Microbiology, 34(2), 360–368. 10.1016/j.fm.2013.01.009 23541203

[fsn32174-bib-0024] Lhomme, E. , Lattanzi, A. , Dousset, X. , Minervini, F. , De Angelis, M. , Lacaze, G. , Onno, B. , & Gobbetti, M. (2015). Lactic acid bacterium and yeast microbiotas of sixteen French traditional sourdoughs. International Journal of Food Microbiology, 215, 161–170. 10.1016/j.ijfoodmicro.2015.09.015 26439422

[fsn32174-bib-0025] Li, X. R. , Ma, E. B. , Yan, L. Z. , Meng, H. , Du, X. W. , & Quan, Z. X. (2013). Bacterial and fungal diversity in the starter production process of Fen liquor, a traditional Chinese liquor. Journal of Microbiology, 51(4), 430–438. 10.1007/s12275-013-2640-9 23990293

[fsn32174-bib-0026] Liu, W. , Zheng, Y. , Kwok, L. Y. , Sun, Z. , Zhang, J. , Guo, Z. , Hou, Q. , Menhe, B. , & Zhang, H. (2015). High‐throughput sequencing for the detection of the bacterial and fungal diversity in Mongolian naturally fermented cow's milk in Russia. BMC Microbiology, 15, 45–45. 10.1186/s12866-015-0385-9 25887414 PMC4345014

[fsn32174-bib-0027] Lu, Y. , Tan, X. , Lv, Y. , Yang, G. , Chi, Y. , & He, Q. (2020). Physicochemical properties and microbial community dynamics during Chinese horse bean‐chili‐paste fermentation, revealed by culture‐dependent and culture‐independent approaches. Food Microbiology, 85, 103309. 10.1016/j.fm.2019.103309 31500715

[fsn32174-bib-0028] Mandhania, M. H. , Paul, D. , Suryavanshi, M. V. , Sharma, L. , Chowdhury, S. , Diwanay, S. S. , Diwanay, S. S. , Shouche, Y. S. , & Patole, M. S. (2019). Diversity and succession of microbiota during fermentation of the traditional Indian food Idli. Applied and Environmental Microbiology, 85(13), e00368‐19. 10.1128/AEM.00368-19 31053581 PMC6581174

[fsn32174-bib-0029] Mathara, J. M. , Schillinger, U. , Kutima, P. M. , Mbugua, S. K. , & Holzapfel, W. H. (2004). Isolation, identification and characterization of the dominant microorganisms of kule naoto: The Maasai traditional fermented milk in Kenya. International Journal of Food Microbiology, 94(3), 269–278. 15246238 10.1016/j.ijfoodmicro.2004.01.008

[fsn32174-bib-0030] Mendoza, L. M. , de Nadra, M. C. M. , & Farías, M. E. (2010). Antagonistic interaction between yeasts and lactic acid bacteria of oenological relevance: Partial characterization of inhibitory compounds produced by yeasts. Food Research International, 43(8), 1990–1998. 10.1016/j.foodres.2010.05.017

[fsn32174-bib-0031] Nahidul‐Islam, S. M. , Kuda, T. , Takahashi, H. , & Kimura, B. (2018). Bacterial and fungal microbiota in traditional Bangladeshi fermented milk products analysed by culture‐dependent and culture‐independent methods. Food Research International, 111, 431–437. 10.1016/j.foodres.2018.05.048 30007706

[fsn32174-bib-0032] Oki, K. , Dugersuren, J. , Demberel, S. , & Watanabe, K. (2014). Pyrosequencing analysis of the microbial diversity of airag, khoormog and tarag, traditional fermented dairy products of mongolia. Bioscience of Microbiota Food and Health, 33(2), 53–64. 10.12938/bmfh.33.53 PMC4081183 25003019

[fsn32174-bib-0033] Patel, A. K. , Singhania, R. R. , Pandey, A. , Joshi, V. K. , Nigam, P. S. , & Soccol, C. R. (2014). Enterobacteriaceae, Coliforms and E. Coli | Introduction. In C. A. Batt , & M. L. Tortorello (Eds.), Encyclopedia of food microbiology (2nd edn, pp. 659–666). Academic Press.

[fsn32174-bib-0034] Pruesse, E. , Quast, C. , Knittel, K. , Fuchs, B. M. , Ludwig, W. , Peplies, J. , & Glockner, F. O. (2007). SILVA: A comprehensive online resource for quality checked and aligned ribosomal RNA sequence data compatible with ARB. Nucleic Acids Research, 35(21), 7188–7196. 10.1093/nar/gkm864 17947321 PMC2175337

[fsn32174-bib-0035] Rodrigues, A. G. (2016). Chapter 6 ‐ Secondary Metabolism and Antimicrobial Metabolites of Aspergillus. In V. K. Gupta (Ed.), New and future developments in microbial biotechnology and bioengineering (pp. 81–93). Elsevier.

[fsn32174-bib-0036] Shangpliang, H. N. J. , Rai, R. , Keisam, S. , Jeyaram, K. , & Tamang, J. P. (2018). Bacterial community in naturally fermented milk products of Arunachal Pradesh and Sikkim of India analysed by high‐throughput amplicon sequencing. Scientific Reports, 8(1), 1532. 10.1038/s41598-018-19524-6 29367606 PMC5784140

[fsn32174-bib-0037] Shangpliang, H. N. , Sharma, S. , Rai, R. , & Tamang, J. P. (2017). Some Technological properties of lactic acid bacteria isolated from Dahi and Datshi, naturally fermented milk products of Bhutan. Frontiers in Microbiology, 8, 116. 10.3389/fmicb.2017.00116 28203227 PMC5285335

[fsn32174-bib-0038] Sieuwerts, S. , Bron, P. A. , & Smid, E. J. (2018). Mutually stimulating interactions between lactic acid bacteria and *Saccharomyces cerevisiae* in sourdough fermentation. LWT, 90, 201–206. 10.1016/j.lwt.2017.12.022

[fsn32174-bib-0039] Song, A. A. , In, L. L. A. , Lim, S. H. E. , & Rahim, R. A. (2017). A review on *Lactococcus lactis*: From food to factory. Microbial Cell Factories, 16(1), 55. 10.1186/s12934-017-0669-x 28376880 PMC5379754

[fsn32174-bib-0040] Sudun, X. , Wulijideligen, X. , Arakawa, K. , Miyamoto, M. , & Miyamoto, T. (2013). Interaction between lactic acid bacteria and yeasts in airag, an alcoholic fermented milk. Animal Science Journal, 84(1), 66–74. 10.1111/j.1740-0929.2012.01035.x 23302085

[fsn32174-bib-0041] Sun, Z. , Liu, W. , Bao, Q. , Zhang, J. , Hou, Q. , Kwok, L. , Sun, T. , & Zhang, H. (2014). Investigation of bacterial and fungal diversity in tarag using high‐throughput sequencing. Journal of Dairy Science, 97(10), 6085–6096. 10.3168/jds.2014-8360 25129502

[fsn32174-bib-0042] Takeda, S. , Yamasaki, K. , Takeshita, M. , Kikuchi, Y. , Tsend‐Ayush, C. , Dashnyam, B. , Ahhmed, A. M. , Kawahara, S. , & Muguruma, M. (2011). The investigation of probiotic potential of lactic acid bacteria isolated from traditional Mongolian dairy products. Animal Science Journal, 82(4), 571–579. 10.1111/j.1740-0929.2011.00874.x 21794017

[fsn32174-bib-0043] Thrane, U. (2014). Fusarium. In C. A. Batt , & M. L. Tortorello (Ed.), Encyclopedia of food microbiology (2nd edn, pp. 76–81). Academic Press.

[fsn32174-bib-0044] Wang, J. , Wu, T. , Fang, X. , Min, W. , & Yang, Z. (2018). Characterization and immunomodulatory activity of an exopolysaccharide produced by *Lactobacillus plantarum* JLK0142 isolated from fermented dairy tofu. International Journal of Biological Macromolecules, 115, 985–993. 10.1016/j.ijbiomac.2018.04.099 29684452

[fsn32174-bib-0045] Wang, Q. , Garrity, G. M. , Tiedje, J. M. , & Cole, J. R. (2007). Naive Bayesian classifier for rapid assignment of rRNA sequences into the new bacterial taxonomy. Applied and Environmental Microbiology, 73(16), 5261–5267. 17586664 10.1128/AEM.00062-07 PMC1950982

[fsn32174-bib-0046] Wang, Y. , Zhou, J. , Xia, X. , Zhao, Y. , & Shao, W. (2016). Probiotic potential of *Lactobacillus paracasei* FM‐LP‐4 isolated from Xinjiang camel milk yoghurt. International Dairy Journal, 62, 28–34. 10.1016/j.idairyj.2016.07.001

[fsn32174-bib-0047] Witthuhn, R. C. , Schoeman, T. , & Britz, T. J. (2004). Isolation and characterization of the microbial population of different South African kefir grains. International Journal of Dairy Technology, 57(1), 33–37. 10.1111/j.1471-0307.2004.00126.x

[fsn32174-bib-0048] Xiao, Y. , Xiong, T. , Peng, Z. , Liu, C. , Huang, T. , Yu, H. , & Xie, M. (2018). Correlation between microbiota and flavours in fermentation of Chinese Sichuan Paocai. Food Research International, 114, 123–132. 10.1016/j.foodres.2018.06.051 30361008

[fsn32174-bib-0049] Yamei, Y. , Guo, Y.‐S. , Zhu, J.‐J. , Xiao, F. , Hasiqimuge, S. , Sun, J.‐P. , Qian, J.‐P. , Xu, W.‐L. , Li, C.‐D. , & Guo, L. (2019). Investigation of physicochemical composition and microbial communities in traditionally fermented vrum from Inner Mongolia. Journal of Dairy Science, 102(10), 8745–8755. 10.3168/jds.2019-16288 31400900

[fsn32174-bib-0050] Yao, G. , Yu, J. , Hou, Q. , Hui, W. , Liu, W. , Kwok, L. Y. , Menghe, B. , Sun, T. , Zhang, H. , & Zhang, W. (2017). A perspective study of koumiss microbiome by metagenomics analysis based on single‐cell amplification technique. Frontiers in Microbiology, 8, 165. 10.3389/fmicb.2017.00165 28223973 PMC5293792

[fsn32174-bib-0051] Yi, L. , Dang, Y. , Wu, J. , Zhang, L. , Liu, X. , Liu, B. , Zhou, Y. , & Lu, X. (2016). Purification and characterization of a novel bacteriocin produced by *Lactobacillus crustorum* MN047 isolated from koumiss from Xinjiang, China. Journal of Dairy Science, 99(9), 7002–7015. 10.3168/jds.2016-11166 27423943

[fsn32174-bib-0052] Yu, J. , Wang, W. H. , Menghe, B. L. , Jiri, M. T. , Wang, H. M. , Liu, W. J. , Bao, Q. H. , Lu, Q. , Zhang, J. C. , Wang, F. , Xu, H. Y. , Sun, T. S. , & Zhang, H. P. (2011). Diversity of lactic acid bacteria associated with traditional fermented dairy products in Mongolia. Journal of Dairy Science, 94(7), 3229–3241. 10.3168/jds.2010-3727 21700007

[fsn32174-bib-0053] Zhang, Y. , Skaar, I. , Sulyok, M. , Liu, X. , Rao, M. , & Taylor, J. W. (2016). The microbiome and metabolites in fermented Pu‐erh tea as revealed by high‐throughput sequencing and quantitative multiplex metabolite analysis. PLoS One, 11(6), e0157847. 10.1371/journal.pone.0157847 27337135 PMC4918958

