# Supplementary material for: A snapshot study of the microbial community dynamics in naturally fermented cow’s milk
Source: Food Sci Nutr. 2021 Feb 16;9(4):2053–65. doi: 10.1002/fsn3.2174 (PMC8020932; doi:10.1002/fsn3.2174)
Supplement: Supplementary file 1 — Table S1 [file FSN3-9-2053-s001.docx]

Supplemental Table S1. The summary of the studies on naturally fermented milk products from different countries.

|  | Sampling location | Production process | Physicochemical composition | Microbial community | Health benefits |
| --- | --- | --- | --- | --- | --- |
| Akabanda et al., 2013 | Ghana | ○ | ○ | ● | ○ |
| Bornaz et al., 2010 | Tunisia | ○ | ● | ○ | ○ |
| Gao et al., 2017 | China | ○ | ○ | ● | ○ |
| Gesudu et al., 2016 | China | ○ | ○ | ● | ○ |
| Guo et al., 2019a | China | ○ | ● | ● | ○ |
| Guo et al., 2019b | China | ● | ● | ○ | ○ |
| Karami et al., 2017 | Iran | ○ | ○ | ○ | ● |
| Liu et al., 2015 | Russia | ○ | ○ | ● | ○ |
| Mathara et al., 2004 | Kenya | ○ | ○ | ● | ○ |
| Nahidul-Islam et al., 2018 | Bangladesh | ○ | ○ | ● | ○ |
| Oki et al., 2014 | Mongolia | ○ | ○ | ● | ○ |
| Shangpliang et al., 2018 | India | ○ | ○ | ● | ○ |
| Shangpliang et al., 2017 | Bhutan | ○ | ○ | ● | ○ |
| Sun et al., 2014 | China Mongolia | ○ | ○ | ● | ○ |
| Takeda et al., 2011 | Mongolia | ○ | ○ | ● | ● |
| Wang et al., 2018 | China | ○ | ○ | ○ | ● |
| Wang et al., 2016 | China | ○ | ○ | ○ | ● |
| Yamei et al., 2019 | China | ● | ● | ● | ○ |
| Yao et al., 2017 | China Mongolia | ○ | ○ | ● | ○ |
| Yi et al., 2016 | China | ○ | ○ | ○ | ● |
| Yu et al., 2011 | Mongolia | ○ | ○ | ● | ○ |
